# Hydrazine Hydrate Induced Three-Dimensional Interconnected Porous Flower-like 3D-NiCo-SDBS-LDH Microspheres for High-Performance Supercapacitor

**DOI:** 10.3390/ma15041405

**Published:** 2022-02-14

**Authors:** Liping Zhong, Zumiao Yan, Hai Wang, Linjiang Wang

**Affiliations:** 1 College of Material Science and Engineering, Guilin University of Technology, Guilin 541004, China; zlp19891230@glut.edu.cn (L.Z.); yzm1592022@163.com (Z.Y.); 2 College of Physics and Technology, Guangxi Normal University, Guilin 541004, China; hbwanghai@gmail.com; 3 Collaborative Innovation Center for Exploration of Hidden Nonferrous Metal Deposits and Development of New Materials in Guangxi, Guilin University of Technology, Guilin 541004, China

**Keywords:** layered double hydroxides, supercapacitors, hydrazine hydrate, surface defects

## Abstract

Porous structure and surface defects are important to improve the performance of supercapacitors. In this study, a facile pathway was developed for high-performance supercapacitors, which can produce transition metal hydroxides (LDHs) with abundant porous structure and surface defects. The NiCo-SDBS-LDH was prepared by one-step hydrothermal reaction using sodium dodecylbenzene sulfonate (SDBS) as anionic surfactant. And then, three dimensional (3D) interconnected porous flower-like 3D-NiCo-SDBS-LDH microspheres were designed and synthesized using the gas-phase hydrazine hydrate reduction method. Results showed that the hydrazine hydrate reduction not only introduces a large number of pores into 3D-NiCo-SDBS-LDH microspheres and causes the formation of oxygen vacancies, but it also roughens the surface of the microspheres. All these changes contribute to the enhancement of electrochemical activity of 3D-NiCo-SDBS-LDH; the NiCo-SDBS-LDH electrode after hydrazine hydrate treatment (3D-NiCo-SDBS-LDH) exhibits a higher specific capacitance of 1148 F·g^−1^ at 1 A·g^−1^ (about 1.46 times larger than that of NiCo-SDBS-LDH) and excellent long cycle life with 94% retention after 4000 cycles. Moreover, the assembled 3D-NiCo-SDBS-LDH//AC (active carbon) asymmetric supercapacitor (ASC) achieves remarkable energy density of 73.14 W h·kg^−1^ at 800 W·kg^−1^ and long-term cycling stability of 95.5% retention for up to 10,000 cycles. The outstanding electrochemical performance can be attributed to the synergy between the rich porous structure and the roughened surface that has been created by the hydrazine hydrate treatment.

## 1. Introduction

Supercapacitors have received a great deal of attention as an energy storage device in the electric vehicle and consumer electronics industries because of their fast charge–discharge features, long cycle stability, and high power densities [1,2,3,4,5]. However, due to their low energy density, they are unable to meet the demands of high-performance supercapacitors in the market [6,7]. As a result, high-performance electrodes are urgently required. 

A significant amount of attention has been focused on the transition metal hydroxides (LDHs), particularly cobalt-based LDHs, due to their rich morphology, low cost, and high activity [8,9]. For example, NiCo-LDH had superior high theoretical specific capacitance of 774 F·g^−1^ at the current density of 0.2 A·g^−1^ [10]. However, the relatively sluggish electrochemical reaction kinetics of NiCo-LDH, like the relatively low charge transport rate, the low specific surface area, poor conductivity, and limited exposed electroactive sites, consequently, hinders the electrochemical performance [11,12,13].

The energy storage mechanism of the supercapacitor depends on the faradaic reactions and charge accretion [14]. Double-layer capacitance and pseudocapacitor are two important storage principles to determine the capacitance of a supercapacitor. However, pseudo-capacitive material based on LDHs has a larger capacitance per gram than double-layer capacitors [15]. Aside from the choice of LDH materials, the nanostructure of LDHs plays an important role in facilitating ion transport within the electrode materials [16,17]. Based on the aforementioned issues, hierarchically porous materials and other hierarchical structures can help to improve superior specific capacitance and energy density, which is thought to be a viable strategy to improve NiCo-LDH electrochemical performance. For instance, NiCo-LDHs with desirable morphologies, e.g., nanosheets [18], nanowires [19], nanoflakes [20], nanotubes [21], nanoflowers [22], and microspheres [23], have been synthesized to improve the electrochemical activity by exposing more active sites and shortening the diffusion lengths of both electrons and ions. In addition, creating surface defects is also an efficient strategy to change the nanostructure of LDHs, which has the potential to improve the activity of electrode materials. In particular, the improvement in intrinsic conductivity can accelerate the diffusion of ions and promote charge transfer dynamics, resulting in enhancement of electrochemical performance [24,25,26,27].

Several modification methods, such as thermal annealing treatment [28,29,30], acid etching [31] or alkali etching [32], NaBH_4_ reduction treatment [26,33,34], and hydrazine hydrate reduction treatment [24,25,35], have been reported in previous studies to generate the defective structures in electrode materials. Among these methods, hydrazine hydrate as a mild reducer has received considerable attention. For example, Li et al. adjusted the generation of oxygen vacancies in Co_3_O_4_ electrodes through controlling the hydrazine hydrate volume to optimize the electrochemical lithium storage [24]. In order to improve the catalytic performance of Co_3_O_4_ nanosheets for rechargeable Li-O_2_ batteries, Gao et al. used hydrazine hydrate reduction to control the production of oxygen vacancies and roughen the surface of Co_3_O_4_ nanosheets [25]. In addition, hydrazine hydrate reduction was also employed by Chen et al. to increase the activity of the oxygen evolution reaction [35]. Thus, hydrazine hydrate as a reducing agent has obvious advantages in manufacturing surface defects and oxygen vacancies for transition metal oxides. However, it should be noted that the above-mentioned methods mainly adopt the “open” system of liquid-phase treatment. Although hydrazine hydrate is a strong polar compound and can be well mixed with water, the surface defects of oxides are affected by the concentration of hydrazine hydrate and other factors. 

Compared to the modification method of gas-phase treatment, liquid-phase treatment has the disadvantages of lengthy cleaning and drying operations, as well as a complex preparation process. In our previous work, we used the solvent vapor to process oxides via a novel “sauna reaction”, and successfully synthesized MoO_3−x_ nanobelts with rich oxygen vacancies [36]. To this end, considering the reductive characteristics of hydrazine hydrate and the low boiling point temperature, combined with our previous research, we consider using the steam of hydrazine hydrate to directly treat the electrode surface. 

In this paper, we present a simple vacuum-assisted hydrazine hydrate reduction strategy for the production of 3D flower-like NiCo-LDH microspheres which are enriched in pores and have surface defects. As expected, the obtained 3D-NiCo-SDBS-LDH electrode delivered an enhanced specific capacitance of 1148 F·g^−1^ at 1 A·g^−1^, as well as long-term stability with a capacitance retention of 94% after 4000 cycles. Moreover, the assembled 3D-NiCo-SDBS-LDH//AC device presented a maximal energy density of 73.14 W h·kg^−1^ at 800 W·kg^−1^ and remarkable cycling stability of 95.5% capacitance retention after 10,000 cycles. Results show that LDH improves electrochemical performance and cycling stability by altering electrode structure of the nanosheets.

## 2. Experimental

### 2.1. Material Preparation

**Scheme 1:**
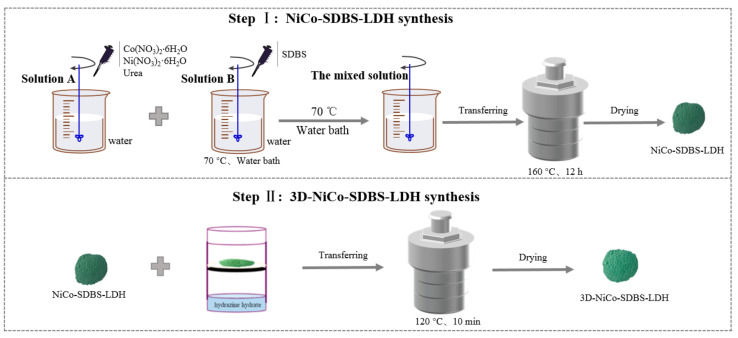
Diagram of the synthesis of 3D-NiCo-SDBS-LDH.

The 3D-NiCo-SDBS-LDH were prepared through a combined strategy, as illustrated in Scheme 1.

Step I: Synthesis of NiCo-SDBS-LDH

According to our prior procedure, the NiCo-SDBS-LDH was prepared by one-step hydrothermal reaction using sodium dodecylbenzene sulfonate (SDBS) as anionic surfactant [37]. Briefly, 6 mmol Ni(NO_3_)_2_·6H_2_O, 2 mmol Co(NO_3_)_2_·6H_2_O, and 20 mmol urea were added into 40 ml de-ionized (DI) water under vigorous magnetic stirring at room temperature for 15 min to obtain solution A.

Meanwhile, solution B was prepared by dissolving 0.4 mmol SDBS in 20 ml DI water and stirring it continuously for 15 min at 70 °C. Following that, the produced solution A was poured into solution B while continuously stirring at 70 °C for 15 min. Then, the mixed solution was put into a stainless-steel Teflon-lined autoclave with a capacity of 100 ml and heated in an oven at 160 °C for 12 h. After being naturally cooled down to room temperature, the precipitates were centrifugated and washed three times by DI water and ethanol. The final products (labeled as NiCo-SDBS-LDH) were collected after drying at 70 °C for 12 h.

Step II: Synthesis of 3D-NiCo-SDBS-LDH

The 3D-NiCo-SDBS-LDH was synthesized using a vacuum-assisted method. The as-prepared NiCo-SDBS-LDH powder was exposed to the hydrazine hydrate vapor at 120 °C for 10 minutes in a 100 ml Teflon-lined stainless steel autoclave, and then cooled to room temperature. After drying at 70 °C for 12 h, the final product labeled as 3D-NiCo-SDBS-LDH was obtained. 

In this study, Ni(NO_3_)_2_·6H_2_O, Co(NO_3_)_2_·6H_2_O, and sodium dodecylbenzene sulfonate (SDBS) were purchased from Alfa Aesar (Shanghai, China). The other chemicals were of EP grade and purchased from Sinopharm Chemical Reagent Co., Ltd. (Shanghai, China).

### 2.2. Structure Characterization

X-ray diffraction (XRD) (Bruker, Billerica, MA, USA) was used to determine the phase composition and crystal structure of the materials using a PANanalytic X’Pert spectrometer (Cu Kα radiation, λ = 0.15405 nm) (Malvern Panalytical, Almelo, the Netherlands). JSM-6300 Field emission scanning electron microscopy (FESEM) (JEOL, Tokyo, Japan), was used to examine the products’ morphologies, and JEM-2010F high resolution transmission electron microscopy (HRTEM) (JEOL, Tokyo, Japan) operating at 200 KV was used to determine additional microstructure characteristics such as crystallinity and morphologies. X-ray photoelectron spectroscopic (XPS) (Bruker, Billerica, MA, USA) examination was performed on materials to analyze surface composition and chemical environment using VG ESCALAB 220 XL (Thermo Scientific, Waltham, MA, USA) instrument at Al Kα (1486.5 eV) radiation. N_2_ adsorption–desorption tests were performed on the two LDH samples for the specific surface area and pore size distribution analysis using a TristarII 3020 analyzer (Micromeritics, Norcross, GA, USA). The UV–Vis spectra were recorded by using a UV–Vis–NIR spectrophotometer (Shimadzu, Kyoto, Japan). The wavelengths of this study were 200–800 nm.

### 2.3. Electrochemical Measurements

The electrochemical performance was measured on an CHI 760E electrochemical workstation (Chenhua, Shanghai, China). The electrochemical performances of the two LDH samples were studied in a three-electrode configuration with Hg/HgO as the reference electrode and platinum as the counter electrode. The working electrodes were fabricated as follows: a mixture of active material, acetylene black, and polyvinylidene fluoride (PVDF) at a weight ratio of 8:1:1 was uniformly cast on nickel foam. This served as the working electrode with an area of 1 cm^2^, and the electrodes were then dried at 90 °C for 10 h in air. Finally, the working electrodes with a loading mass of about 1.68 mg were prepared. The cyclic voltammogram (CV) and galvanostatic charge–discharge (GCD) were tested in voltage window within 0–0.7 V and 0–0.55 V, respectively. Electrochemical impedance spectroscopy (EIS) (Chenhua, Shanghai, China,) was carried out using open-circuit voltage from 0.01 to 100 kHz. 

All electrochemical experiments were performed in 1 M KOH solution on a CHI 760E electrochemical workstation. The electrode’s specific capacitance Cs (F·g^−1^) was calculated using Equation (1) [38]:

(1) $$C_{s}=\frac{I\times \Delta t}{m\times \Delta V}$$

where *I* is the discharge current, A; ∆*t* is the discharge time, s; *m* is the mass loading of the active material, g; and Δ*V* is the potential window, V.

### 2.4. Electrochemical Measurements of ASC Device

The ASC device was built in a two-electrode system with the as-prepared product and activated carbon (AC) acting as positive and negative electrodes, respectively. To obtain charge balance, the positive (+) and negative (−) electrode weight ratios were computed using the Equation (2).

(2) $$\frac{m^{+}}{m^{-}}=\frac{C^{-}\times \Delta V^{-}}{C^{+}\times \Delta V^{+}}$$

where *m* represents the mass loading, g; *C* is the specific capacitance, F·g^−1^; and ∆*V* is the potential window, V. Specific capacitance *C_s_* (F·g^−1^) was determined by Formula (3).

(3) $$C_{s}=\frac{I\times \Delta t}{m^{\prime }\times \Delta V^{\prime }}$$

where *I* is current, A; ∆*t* is the time of discharge process, s; *m*′ is the total weight of the two electrodes, g; and ∆*V*′ is the operating voltage window, V.

Equations (4) and (5) were used to calculate the, energy density and power density, respectively [39,40].

(4) $$E=\frac{I\times \int Vdt}{M}$$

(5) $$P=\frac{E}{t}$$

where *E* is energy density, W h·kg^−1^; *M* is the total active mass of both electrodes, kg; *P* is power density, W·kg^−1^; *t* is the time of discharge process, s; and ∫*Vdt* is the galvanostatic discharge current area.

## 3. Results and Discussion

**Figure 1:**
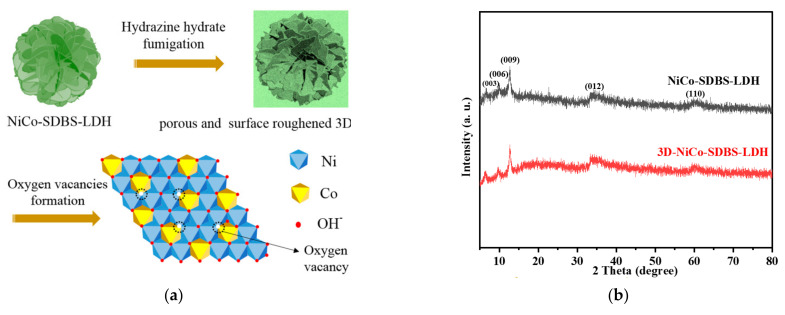
(**a**) Hydrazine hydrate fumigation induced surface structure change and the formation of oxygen vacancies. (**b**) The XRD patterns of the samples before and after hydrazine hydrate treatment.

In this study, hydrazine hydrate was selected as the reducing medium. It has a mild reduction capacity and the reductive by-products such as nitrogen and water are harmless. The preparation process of 3D-NiCo-SDBS-LDH is shown in Figure 1a. The reduction capacity of hydrazine hydrate can help to produce abundant pores, roughen the surfaces of NiCo-SDBS-LDH nanosheets, and induce the formation of oxygen vacancies. These results are consistent with those of Li et al. [24] who use hydrazine hydrate as the reduction medium to modify the structure of Co_3_O_4_ electrodes and improve the electrochemical lithium storage properties. Similar results were also reported by Hazra et al. [41], who adjusted the number of oxygen vacancies in TiO_2_ nanotubes by hydrazine hydrate to improve the storage performance. In addition, hydrazine hydrate reduction was employed by Gakhar et al. [42] as a low-cost and relatively stable method to modify the titania nanotubes.

As shown in Figure 1a, the enriched pores and surface defects contribute to providing more active sites and improving the conductivity of the electrode materials. Changes of microstructure can significantly improve the electrochemical activity [25,43,44,45].

The structures of the as-prepared NiCo-SDBS-LDH and 3D-NiCo-SDBS-LDH were investigated by XRD, as shown in Figure 1b. The XRD patterns of as-synthesized samples exhibited unique (003), (006), (009), (012), and (110) typical diffraction planes. Such results suggest that the as-obtained LDH samples were successfully prepared, and the type of LDH layer anion was SDBS (FTIR spectra is in Figure S1 of Supplementary Material) [37]. In addition, the LDH framework structure was not changed by fumigation of hydrazine hydrate. On the XRD pattern of 3D-NiCo-SDBS-LDH, the diffraction peaks at 2θ values of 6.05°, 9.11°, and 33.48° could be indexed to (003), (006), and (012) crystal planes of LDH, respectively. The corresponding d values were d(003) = 1.54 nm, d(006) = 0.98 nm, and d(012) = 0.27 nm, respectively. On the NiCo-SDBS-LDH spectrum, the peaks at 2θ values of 6.8°, 9.98°, and 34.61° corresponded to the internal spacing of d(003) = 1.28 nm, d(006) = 0.89 nm, and d(012) = 0.25 nm, respectively. It was found that the XRD characteristic diffraction peaks of 3D-NiCo-SDBS-LDH wholly shifted to a smaller angle, indicating the tunable interlayer spacing of LDH. In particular, the d(003) value of the 3D-NiCo-SDBS-LDH increased from 1.28 to 1.54 nm after hydrazine hydrate treatment indicating the expanded interlayer spacing. Obviously, this has a favorable impact on the charge storage capacity of supercapacitors [37]. Furthermore, the crystal plane spacings of NiCo-SDBS-LDH and 3D-NiCo-SDBS-LDH reflected on (012) crystal plane were 0.25 nm and 0.27 nm, respectively, which is good agreement with the following results of HRTEM.

**Figure 2:**
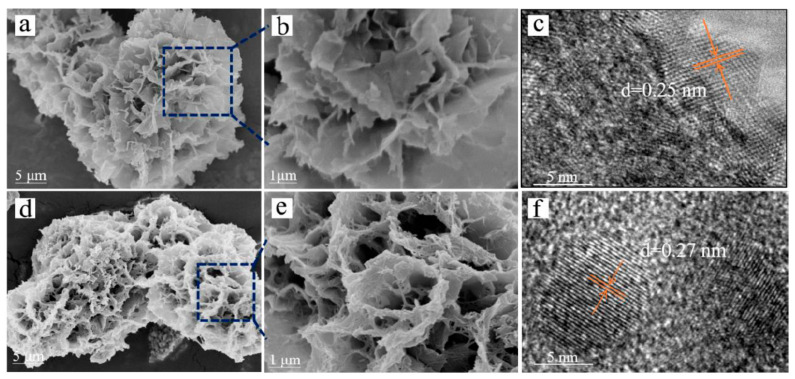
(**a**,**b**) SEM of NiCo-SDBS-LDH at different magnifications; (**d**,**e**) SEM of 3D-NiCo-SDBS-LDH at different magnifications; and (**c**,**f**) HRTEM images of NiCo-SDBS-LDH and 3D-NiCo-SDBS-LDH.

**Figure 3:**
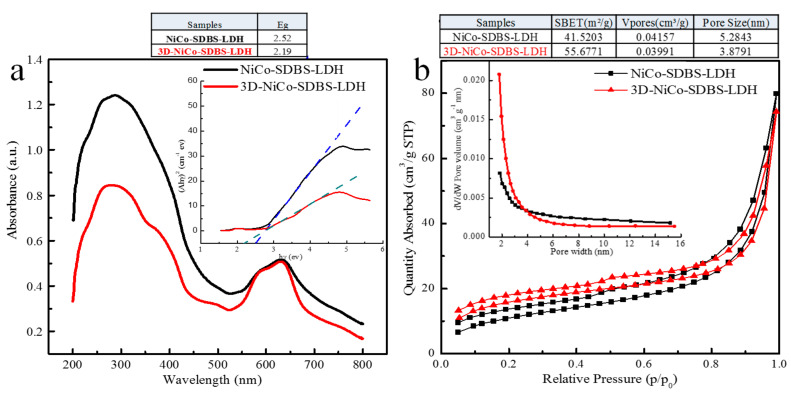
(**a**) UV–Vis spectra, the plots of the (αhv)2 versus photo energy(inset) and (**b**) N_2_ adsorption–desorption isotherms and pore size distribution (inset) for NiCo-SDBS-LDH and 3D-NiCo-SDBS-LDH.

The SEM and TEM images of the samples before and after hydrazine hydrate treatment are shown in Figure 2. The NiCo-SDBS-LDH morphologies at low and high magnifications can be observed by Figure 2a,b, and are composed of nanosheets with smooth surface. Furthermore, the nanosheets are intimately interwoven with one another and interlaced like petals, creating a complex structure. In the case of 3D-NiCo-SDBS-LDH, the SEM images (Figure 2d,e) reveal that the original morphology of NiCo-SDBS-LDH was still maintained. Importantly, the nanosheets gradually transferred into abundant porous structures and displayed a rough and discontinuous surface under the vacuum-assistance of hydrazine hydrate. On the one hand, the porous structure increases the area of the electrode in contact with the electrolyte [24]. On the other hand, a rough surface can increase the number of active sites and conductivity [25]. Undoubtedly, the 3D-NiCo-SDBS-LDH will exhibit a proportionately improved electrochemical performance as a result of these characteristics. HR-TEM micrographs of NiCo-SDBS-LDH and 3D-NiCo-SDBS-LDH are shown in Figure 2c,f, respectively. The crystal planes (012) of NiCo-SDBS-LDH and 3D-NiCo-SDBS-LDH can be clearly indexed with lattice spacings of 0.25 and 0.27 nm, showing that the interlayer spacing was increased following hydrazine hydrate reduction, providing advantageous paths for fast electron transport. Furthermore, 3D-NiCo-SDBS-LDH had a smaller bandgap (Figure 3a), and the surface area increased from 41.52 m^2^·g^−1^ to 55.68 m^2^·g^−1^ (Figure 3b), indicating that after hydrazine hydrate reduction, electron transportation and the material’s electrical conductivity and electroactive surface area were all facilitated.

**Figure 4:**
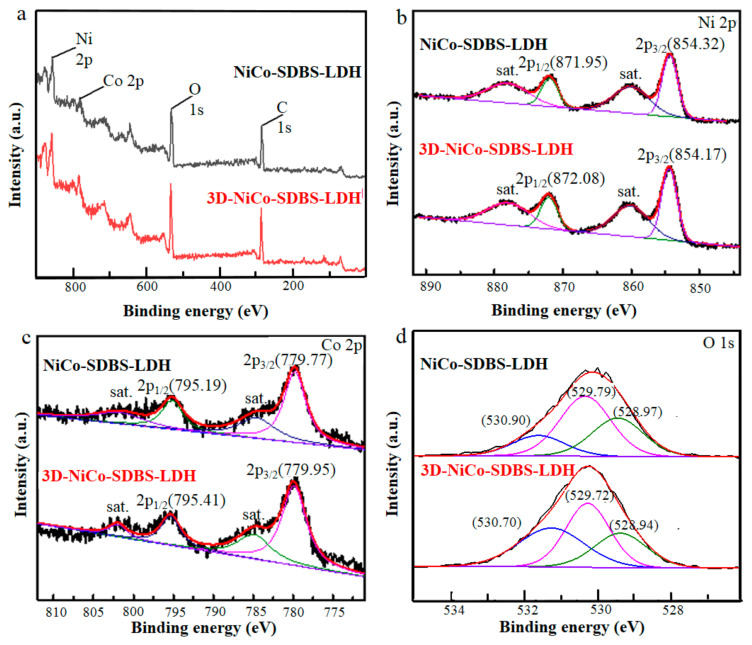
(**a**) XPS survey spectra. High-resolution spectra of (**b**) Ni 2p, (**c**) Co 2p, and (**d**) O 1s of NiCo-SDBS-LDH and 3D-NiCo-SDBS-LDH.

As illustrated in Figure 4, XPS analysis was used to provide further information on the valence state of the elements and the chemical compositions of the two LDH samples.

The high-resolution Ni 2p and Co 2p spectra of NiCo-SDBS-LDH in Figure 4b,c displayed two main peaks and two satellite peaks (denoted as Sat.). The peaks of Ni 2p1/2 and Ni 2p3/2 located at 854.32 eV and 871.95 eV corresponded to Ni^2+^ [36]. Similarly, in the Co 2p spectra, the binding energies at 779.77 eV and 795.19 eV belonged to Co^3+^. In comparison to NiCo-SDBS-LDH, the peak location of 3D-NiCo-SDBS-LDH was slightly shifted and the powder color changed (Scheme 1), showing that the Ni and Co coordination environments had been altered during hydrazine hydrate etching. The O 1s spectra for the two samples are shown in Figure 4d. The binding energies of NiCo-SDBS-LDH centered at 530.90 eV corresponding to oxygen defects [36]. The increase and the higher ratio of defect sites’ binding intensity demonstrating that more oxygen vacancies and surface defects existed in the 3D-NiCo-SDBS-LDH sample after the gas-phase modification with hydrazine hydrate.

**Figure 5:**
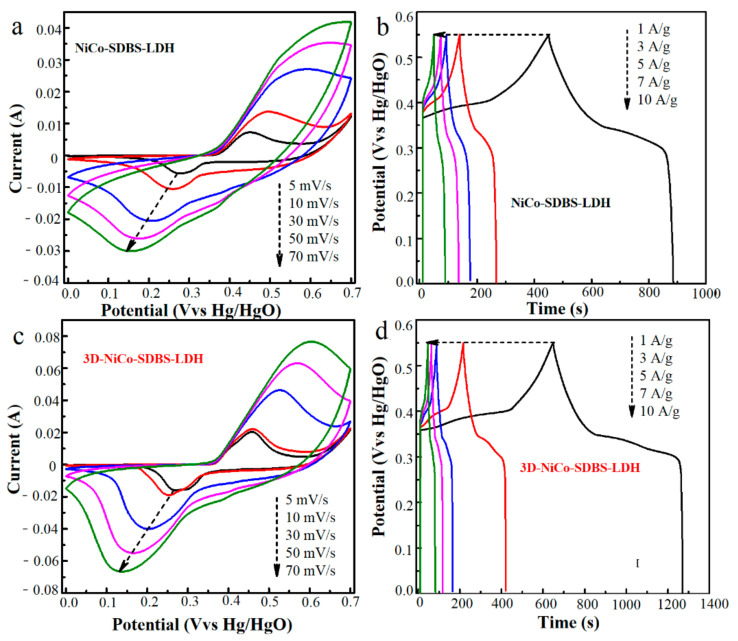
(**a**) CV curves and (**b**) GCD curves of NiCo-SDBS-LDH at different scan rates and current densities, respectively. (**c**) CV curves and (**d**) GCD curves of 3D-NiCo-SDBS-LDH at different scanning rates and current densities, respectively.

Further, the supercapacitor electrochemical performance of the prepared electrodes was tested via CV, GCD, and EIS in a three-electrode system using 1 M KOH electrolyte. Figure 5a,c are the CV curves of NiCo-SDBS-LDH and 3D-NiCo-SDBS-LDH electrodes at various scan rates of 5, 10, 30, 50, and 70 mV·s^−1^ in a potential range of 0.0 to 0.7 V. When the scan rate was increased from 5 to 70 mV·s^−1^, the shape of CV curves remained unchanged, indicating the good rate performance of the samples. As the scan rate rose, the current responses increased, demonstrating that the electrode materials had a high degree of electrochemical reversibility. Additionally, it was discovered that the redox peak shifted in a positive or negative direction as a result of electrode polarization. Figure 5b,d illustrates the GCD curves of the two electrodes at current densities of 1, 3, 5, 7, and 10 A·g^−1^. The geometry of the GCD curves at all densities was almost symmetrical and nonlinear, indicating that the electrodes exhibited good electrochemical reversibility and pseudo-capacitive properties.

**Figure 6:**
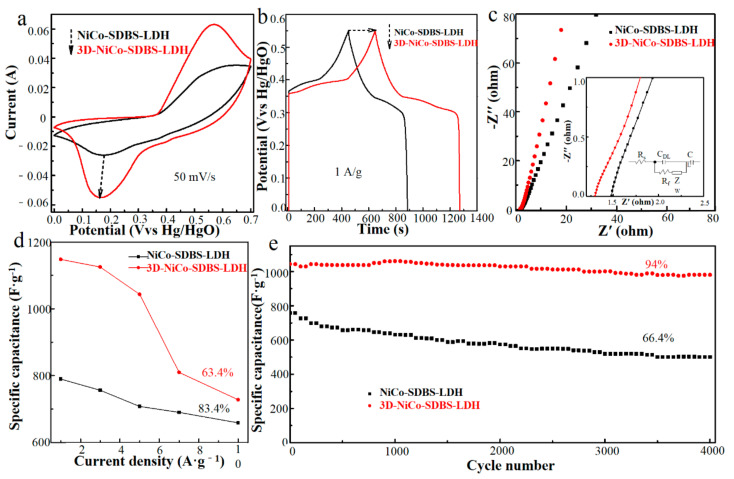
The electrochemical characterizations of as-prepared samples in a three-electrode system. (**a**) CV curves at 50 mV·s^−1^, (**b**) GCD curves at 1 A·g^−1^, (**c**) EIS diagrams, (**d**) specific capacitance at different current density, and (**e**) cycling performances at 5 A·g^−1^ for 4000 cycles of NiCo-SDBS-LDH and 3D-NiCo-SDBS-LDH.

In order to facilitate comparison of the electrochemical behavior of the two electrodes, Figure 6a,b illustrates CV and GCD curves at 50 mV·s^−1^ and 1 A·g^−1^, respectively. As can be seen, the 3D-NiCo-SDBS-LDH electrode had a bigger CV integrated area and a longer charge–discharge time than NiCo-SDBS-LDH, showing superior capacitance performance. The 3D-NiCo-SDBS-LDH had a specific capacitance of 1148 F·g^−1^ at the current density of 1 A·g^−1^, which was significantly greater than 789 F·g^−1^ of NiCo-SDBS-LDH. The enhanced electrochemical performance of the 3D-NiCo-SDBS-LDH electrode can be attributed to the porous structure, which increased the area of contact between the electrode and the electrolyte, and the rough surface, which provided additional active sites and increases conductivity. Compared with other Co-based LDH electrode materials as illustrated in Table 1 [10,32,46,47,48,49,50,51,52,53], such as CoAl-LDH/polypyrrole/graphene (864 F·g^−1^ at 1 A·g^−1^) [48], NiCo-LDH@Ni-CAT nanoflower(882 F·g^−1^ at 1 A·g^−1^) [49], E-CoZnAl-LDH-8 h (946 F·g^−1^ at 1 A·g^−1^) [32], NiCo LDH/3D rGO (1054 F·g^−1^ at 1 A·g^−1^) [50], and α-phase NiCo LDH microsphere (1120 F·g^−1^ at 1 A·g^−1^) [51], our synthesized 3D-NiCo-SDBS-LDH electrode also showed a good charge storage capability.

Additionally, as shown in Figure 6d, the specific capacitance values at various current densities were determined and compared to pristine NiCo-SDBS-LDH. Results show that although the 3D-NiCo-SDBS-LDH electrode only retained 63.4% capacitance at increasing current density from 1 A·g^−1^ to 10 A·g^−1^, it still had a higher specific capacitance than NiCo-SDBS-LDH in the overall trend, this was due to the unique structure and high specific surface area of 3D-NiCo-SDBS-LDH.

It is well-established that the EIS test can provide direct information about the charge transfer of electrodes. Figure 6c illustrates the initial EIS of the two electrodes. The inset of Figure 6c shows the fitting curve and the corresponding equivalent circuit model. 3D-NiCo-SDBS-LDH appears to have a lower charge transfer resistance (R_ct_) than NiCo-SDBS-LDH, implying faster electron transport kinetics and ion diffusion at the electrode/electrolyte interface. Meanwhile, the low-frequency straight line of 3D-NiCo-SDBS-LDH is slanted more toward the Y-axis than that of Ni-Co-SDBS-LDH, showing that protons and electrolyte ions diffused more efficiently, and also indicating that the 3D-NiCo-SDBS-LDH electrode had a lower diffusion resistance (R_w_). The stability performances of the as-synthesized electrodes were then evaluated by repeatedly charging and discharging at a current density of 5 A·g^−1^ (Figure 6e). The 3D-NiCo-SDBS-LDH electrode showed a huge capacitance retention of 94% over 4000 cycles, while the retention rate of the NiCo-SDBS-LDH sample was only 66.4%.

**Figure 7:**
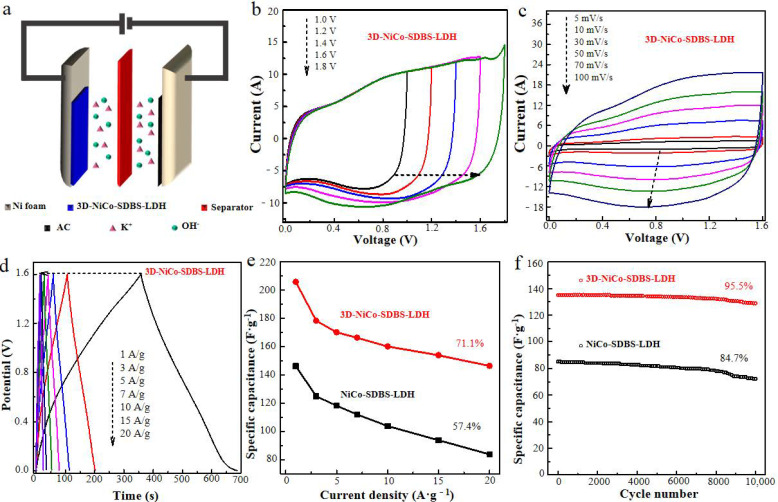
(**a**) Schematic diagram of the assembly ASC devices, (**b**) CV curves with different voltage windows at 50 mV·s^−1^, (**c**) CV curves at different scan rates from 5 to 100 mV·s^−1^, (**d**) GCD curves at different current densities for 3D-NiCo-SDBS-LDH//AC ASCs device, (**e**) specific capacitance at different current density, and (**f**) cycling performance at 5 A·g^−1^ for 10,000 cycles of 3D-NiCo-SDBS-LDH//AC and 3D-NiCo-SDBS-LDH//AC ASCs devices.

To further evaluate the practical applications of the as-prepared electrodes in supercapacitors, the ASC devices were constructed using KOH aqueous solution, the as-prepared electrode, activated carbon and filter paper as the electrolyte, a positive electrode, a negative electrode, and a separator, as shown in Figure 7a.

According to the CV and GCD curves of activated carbon electrode in Figure S2 of the Supplementary Material, the potential window was −1–0 V and the specific capacitance was 186 F·g^−1^ at 1 A·g^−1^. Based on the measurement above and Equation (2), the optimum m+/m− ratio should equal to 0.162 in the as-assembled device. The CV curves of the 3D-NiCo-SDBS-LDH // AC device at different voltage windows at a scan rate of 50 mV·s^−1^ in Figure 7b show that the shapes were observed to remain stable when the voltage window gradually increased from 0–1.0 V to 0–1.6 V, until the voltage window reached 0–1.8 V and the CV curve deformed, revealing that the most optimal voltage window of the device was 0–1.6 V. Figure 7c shows the CV curves of 3D-NiCo-SDBS-LDH//AC at different scan rates from 5 to 100 mV·s^−1^ in the voltage window of 0–1.6 V. The curves demonstrate that as the scan rate increased, the integrated area grew as well, indicating that the capacitive behavior was optimal. The GCD curves at various current densities were measured in order to have a better understanding of the electrochemical performance (Figure 7d); the specific capacitances of the device were estimated as 205.7, 178.1, 170.0, 166.2, 160, 153.8, and 146.3 F·g^−1^ at current densities of 1, 3, 5, 7, 10, 15, and 20 A·g^−1^, respectively. Meanwhile, the related energy and power density of ASCs devices were calculated. The maximum energy density of our fabricated 3D-NiCo-SDBS-LDH//AC device reached as high as 73.14 W h·kg^−1^ at power density of 800 W·kg^−1^, which is higher than many previously reported NiCo-LDH-based ASCs demonstrated in Table 2 [38,54,55,56,57,58]. Remarkably, the 3D-NiCo-SDBS-LDH//AC device retained 71.1% capacitance at increasing current density from 1 A·g^−1^ to 20 A·g^−1^ (Figure 7e), revealing the good rate performance. Simultaneously, for comparison, assembling the NiCo-SDBS-LDH//AC device, the capacitance retention was only 57.4%. Notably, the 3D-NiCo-SDBS-LDH//AC device also exhibited extraordinary long-term life with the capacitance retention of 95.50% over10000 cycles at 5 A·g^−1^ (Figure 7f), which was higher than the 84.7% of NiCo-SDBS-LDH// AC. Such cycling stability outperforms those reported for NiCo-LDH-based ASCs devices such as CoSx/NiCo LDH nanocages//AC (94.56% after 10000 cycles) [54], NiCo-LDH/Ag nanowires//AC (88.2% after 2000 cycles) [55], His-GQD/NiCo-LDH//AC (91.13% after 6000 cycles) [56], and PNT@NiCo-LDH//AC (84.3% after 5000 cycles) [58].

## 4. Conclusions

In summary, the pore-enriched and surface-defected 3D flower-like NiCo-LDH microspheres were developed using the gas-phase hydrazine hydrate reduction method by a simple vacuum-assisted strategy. Due to the porous morphology and the existence of oxygen vacancies, the electrochemical activity of 3D-NiCo-SDBS-LDH was greatly improved.

The obtained 3D-NiCo-SDBS-LDH combined the advantages of abundant porous structure and surface defect, which largely enhanced the activity and stability of the electrode material. Typically, the 3D-NiCo-SDBS-LDH electrode had an excellent capacitive property (1148 F·g^−1^ at 1 A·g^−1^) and great cycling performance (94% capacitance retention after 4000 cycles), compared to 789 F·g^−1^ and 66.4% of NiCo-SDBS-LDH electrode. Furthermore, the as-fabricated 3D-NiCo-SDBS-LDH//AC ASC attained a considerable energy density of 73.14 W h·kg^−1^ (800 W·kg^−1^) and remarkable cycling stability of 95.5% (after 10,000 cycles), which are higher than many previously reported ASCs.

This work provides a simple and effective method to modify the microstructure of transition metal hydroxides (LDHs), which improves the electrochemical performance for different applications.

## Figures and Tables

**Table 1:** Electrochemical property comparison of our as-synthesized 3D-NiCo-SDBS-LDH with Co-based LDH electrodes in some published papers.

Sample	Substrate	Electrolyte	C_s_ (F·g^−1^)	Cycling Stability	Ref.
NiCo-LDH nanosheets	NF	1 M KOH	774 F·g^−1^ at 0.2 A·g^−1^	75% (500)	[10]
E-CoZnAl-LDH-8 h	NF	1 M KOH	946 F·g^−1^ at 1 A·g^−1^	92.3% (4000)	[32]
MnO_2_-2/NiCo-LDH	CC	1 M Na_2_SO_4_	312 F·g^−1^ at 0.2 A·g^−1^	97% (5000)	[46]
NiCoAl-LDH nanosheets	CC	1 M KOH	1137 F·g^−1^ at 0.5 A·g^−1^	100% (12,000)	[47]
CoAl-LDH/polypyrrole/graphene	NF	30 wt% KOH	864 F·g^−1^ at 1 A·g^−1^	100% (5000)	[48]
NiCo-LDH@Ni-CAT nanoflower	/	1 M KOH	882 F·g^−1^ at 1 A·g^−1^	64% (5000)	[49]
NiCo LDH/3D rGO	NF	6 M KOH	1054 F·g^−1^ at 1 A·g^−1^	95% (2000)	[50]
α-phase NiCo LDH microsphere	NF	6 M KOH	1120 F·g^−1^ at 1 A·g^−1^	93.8% (3000)	[51]
MXene-NiCo LDH	TC	6 M KOH	983.6 F·g^−1^ at 2 A·g^−1^	76% (5000)	[52]
KCu_7_S_4_@NiCo LDH	/	/	1104.5 F·g^−1^ at 2 A·g^−1^	83.5% (1000)	[53]
3D-NiCo-SDBS-LDH	NF	1 M KOH	1148 F·g^−1^ at 1 A·g^−1^	94% (4000)	this work

NF: nickel foam; CC: carbon cloth; TC: titanium carbide.

**Table 2:** Energy density comparison of our as-fabricated 3D-NiCo-SDBS-LDH//AC with NiCo-LDH-based ASCs in some published papers.

Positive Electrode	Negative Electrode	Energy Density (W h·kg^−1^)	Cycling Stability	Ref.
NiCo-LDH/ZnO nanoflakes	AC	51.39 W h·kg^−1^ at 800 W·kg^−1^	87.3% (1000)	[38]
CoS_x_/NiCo LDH nanocages	AC	35.8 W h·kg^−1^ at 800 W·kg^−1^	94.56% (10,000)	[54]
NiCo-LDH/Ag nanowires	AC	42.9 W h·kg^−1^ at 800 W·kg^−1^	88.2% (2000)	[55]
His-GQD/NiCo-LDH	AC	48.89 W h·kg^−1^ at 800 W·kg^−1^	91.13% (6000)	[56]
NiCo-LDH/GF/NF	AC	50.2 W h·kg^−1^ at 800 W·kg^−1^	-	[57]
PNT@NiCo-LDH	AC	64.4 W h·kg^−1^ at 800 W·kg^−1^	84.3% (5000)	[58]
3D-NiCo-SDBS-LDH	AC	73.14 W h·kg^−1^ at 800 W·kg^−1^	95.5% (10,000)	this work

## Data Availability

The data sets used and analyzed that support the findings of this study are available from the corresponding author upon reasonable request.

## Supplementary Materials

The following supporting information can be downloaded at: https://www.mdpi.com/article/10.3390/ma15041405/s1, Figure S1: FTIR spectra of NiCo-SDBS-LDH and 3D-NiCo-SDBS-LDH, Figure S2: CV curves at 10 mV·s^−1^ and GCD curves at 1 A·g^−1^ of activated carbon.

## References

[1] Liu Y., Hu P., Liu H., Song J., Umar A., Wu X. (2019). Toward a high performance asymmetric hybrid capacitor by electrode optimization. Inorg. Chem. Front..

[2] Wei B., Mei G., Liang H., Qi Z., Zhang D., Shen H., Wang Z. (2018). Porous CrN thin films by selectively etching CrCuN for symmetric supercapacitors. J. Power Sources.

[3] Li X., Yang X., Xue H., Pang H., Xu Q. (2020). Metal–organic frameworks as a platform for clean energy applications. EnergyChem.

[4] Zhang G., Xiao X., Li B., Gu P., Xue H., Pang H. (2017). Transition metal oxides with one-dimensional/one-dimensional-analogue nanostructures for advanced supercapacitors. J. Mater. Chem. A.

[5] Zhao Y., He J., Dai M., Zhao D., Wu X., Liu B. (2020). Emerging CoMn-LDH@MnO_2_ electrode materials assembled using nanosheets for flexible and foldable energy storage devices. J. Energy Chem..

[6] Lu Y., Li Z., Bai Z., Mi H., Ji C., Pang H., Yu C., Qiu J. (2019). High energy-power Zn-ion hybrid supercapacitors enabled by layered B/N co-doped carbon cathode. Nano Energy.

[7] Wu X., Jiang L., Long C., Wei T., Fan Z. (2015). Dual support system ensuring porous Co-Al hydroxide nanosheets with ultrahigh rate performance and high energy density for supercapacitors. Adv. Funct. Mater..

[8] Han L., Dong S., Wang E. (2016). Transition-Metal (Co, Ni, and Fe)-Based Electrocatalysts for the Water Oxidation Reaction. Adv. Mater..

[9] Dou S., Wang X., Wang S. (2019). Rational Design of Transition Metal-Based Materials for Highly Efficient Electrocatalysis. Small Methods.

[10] Wang C., Zhang X., Sun X., Ma Y. (2016). Facile fabrication of ethylene glycol intercalated cobalt-nickel layered double hydroxide nanosheets supported on nickel foam as flexible binder-free electrodes for advanced electrochemical energy storage. Electrochim. Acta.

[11] Jing C., Huang Y., Xia L., Chen Y., Wang X., Liu X., Dong B., Dong F., Li S., Zhang Y. (2019). Growth of cobalt-aluminum layered double hydroxide nanosheets on graphene oxide towards high performance supercapacitors: The important role of layer structure. Appl. Surf. Sci..

[12] Lee I., Park M.Y., Kim H.J., Lee J.H., Park J.Y., Hong J., Kim K.I., Park M., Yun J.Y., Yoon K.J. (2017). High-Temperature Current Collection Enabled by the in Situ Phase Transformation of Cobalt-Nickel Foam for Solid Oxide Fuel Cells. ACS Appl. Mater. Interfaces.

[13] Wu Z., Khalafallah D., Teng C., Wang X., Zou Q., Chen J., Zhi M., Hong Z. (2020). Vanadium doped hierarchical porous nickel-cobalt layered double hydroxides nanosheet arrays for high-performance supercapacitor. J. Alloys Compd..

[14] Peçenek H., Yetiman S., Dokan F.K., Onses M.S., Yılmaz E., Sahmetlioglu E. (2022). Effects of carbon nanomaterials and MXene addition on the performance of nitrogen doped MnO_2_ based supercapacitors. Ceram. Int..

[15] Lamiel C., Hussain I., Shim J.J. (2017). Enhancement of electrochemical performance of nickel cobalt layered double hydroxide@ nickel foam with potassium ferricyanide auxiliary electrolyte. Energy.

[16] Hasan S.W., Taha Z., Meng Q., Shen J., Lyu T., Zhu J., Shen P.K. (2020). Interstitial nanoclusters within graphene sheets for highly conductive, strong and electrochemically active fiber-shaped supercapacitors. Appl. Mater. Today.

[17] Peçenek H., Dokan F.K., Onses M.S., Yılmaz E., Sahmetlioglu E. (2022). Outstanding Supercapacitor Performance With Intertwined Flower-Like NiO/MnO_2_/CNT Electrodes. Mater. Res. Bull..

[18] Wang X., Li X., Du X., Ma X., Hao X., Xue C., Zhu H., Li S. (2017). Controllable Synthesis of NiCo LDH Nanosheets for Fabrication of High-Performance Supercapacitor Electrodes. Electroanalysis.

[19] Su W., Wu F., Fang L., Hu J., Liu L., Guan T., Long X., Luo H., Zhou M. (2019). NiCo-LDH nanowires@nanosheets core-shell structure grown on carbon fiber cloth for high performance flexible supercapacitor electrode. J. Alloys Compd..

[20] Yue L., Jia D., Tang J., Zhang A., Liu F., Chen T., Barrow C., Yang W., Liu J. (2020). Improving the rate capability of ultrathin NiCo-LDH nanoflakes and FeOOH nanosheets on surface electrochemically modified graphite fibers for flexible asymmetric supercapacitors. J. Colloid Interface Sci..

[21] Zhu F., Liu W., Liu Y., Shi W. (2020). Construction of porous interface on CNTs@NiCo-LDH core-shell nanotube arrays for supercapacitor applications. Chem. Eng. J..

[22] Zhou Y., Li J., Yang Y., Luo B., Zhang X., Fong E., Chu W., Huang K. (2019). Unique 3D flower-on-sheet nanostructure of NiCo LDHs: Controllable microwave-assisted synthesis and its application for advanced supercapacitors. J. Alloys Compd..

[23] Ramachandran R., Lan Y., Xu Z.X., Wang F. (2020). Construction of NiCo-Layered Double Hydroxide Microspheres from Ni-MOFs for High-Performance Asymmetric Supercapacitors. ACS Appl. Energy Mater..

[24] Li Z., Qian L., Chen J., Zhang W., Tian W., Wan Y., Yang P., Wang Z., Liu Y., Huang W. (2021). Hydrazine hydrate reduction-induced oxygen vacancy formation in Co_3_O_4_ porous nanosheets to optimize the electrochemical lithium storage. J. Alloys Compd..

[25] Gao R., Shang Z., Zheng L., Wang J., Sun L., Hu Z., Liu X. (2019). Enhancing the Catalytic Activity of Co_3_O_4_ Nanosheets for Li-O_2_ Batteries by the Incoporation of Oxygen Vacancy with Hydrazine Hydrate Reduction. Inorg. Chem..

[26] Liang H., Jia H., Lin T., Wang Z., Li C., Chen S., Qi J., Cao J., Fei W., Feng J. (2019). Oxygen-vacancy-rich nickel-cobalt layered double hydroxide electrode for high-performance supercapacitors. J. Colloid Interface Sci..

[27] Yang C., Gao R., Yang H. (2021). Application of layered nanoclay in electrochemical energy: Current status and future. EnergyChem.

[28] Liu Y., Zhang H., Wan H., Zhang W., Jiang N., Huang G., Wang Z., Luo S., Sun H. (2019). Tuning lithium storage properties of cubic Co_3_O_4_ crystallites: The effect of oxygen vacancies. J. Alloys Compd..

[29] Liu Y., Zhang H., Jiang N., Zhang W., Arandiyan H., Wang Z., Luo S., Fang F., Sun H. (2020). Porous Co_3_O_4_@CoO composite nanosheets as improved anodes for lithium-ion batteries. J. Alloys Compd..

[30] Liu Y., Wan H., Zhang H., Chen J., Fang F., Jiang N., Zhang W., Zhou F., Arandiyan H., Wang Y. (2020). Engineering Surface Structure and Defect Chemistry of Nanoscale Cubic Co_3_O_4_ Crystallites for Enhanced Lithium and Sodium Storage. ACS Appl. Nano Mater..

[31] Zhou P., Wang Y., Xie C., Chen C., Liu H., Chen R., Huo J., Wang S. (2017). Acid-etched layered double hydroxides with rich defects for enhancing the oxygen evolution reaction. Chem. Commun..

[32] Yang C., Zhang B., Xie X., Li C., Xu Y., Wang H., Wang L. (2020). Three-dimensional independent CoZnAl-LDH nanosheets via asymmetric etching of Zn/Al dual ions for high-performance supercapacitors. J. Alloys Compd..

[33] Liu Y., Wan H., Jiang N., Zhang W., Zhang H., Chang B., Wang Q., Zhang Y., Wang Z., Luo S. (2019). Chemical reduction-induced oxygen deficiency in Co_3_O_4_ nanocubes as advanced anodes for lithium ion batteries. Solid State Ion..

[34] Sun H., Zhao Y., Mølhave K., Zhang M., Zhang J. (2017). Simultaneous modulation of surface composition, oxygen vacancies and assembly in hierarchical Co_3_O_4_ mesoporous nanostructures for lithium storage and electrocatalytic oxygen evolution. Nanoscale.

[35] Chen R., Tan Y., Zhang Z., Lei Z., Wu W., Cheng N., Mu S. (2020). Hydrazine Hydrate Induced Two-dimensional Porous Co^3+^ enriched Co_3_O_4_ nanosheets for Enhanced Water Oxidation Catalysis Hydrazine Hydrate Induced Two-dimensional Porous Co^3+^ enriched Co_3_O_4_ nanosheets for Enhanced Water Oxidation Catalysis. ACS Sustain. Chem. Eng..

[36] Sun Z., Yang C., Liu G., Lu H., Zhang R., Wang L., Wang H. (2017). Largely enhanced electrochemical performance in MoO_3−x_ nanobelts formed by a “sauna reaction”: Importance of oxygen vacancies. Electrochim. Acta.

[37] Lin Y., Xie X., Wang X., Zhang B., Li C., Wang H., Wang L. (2017). Graphical Abstract Understanding the enhancement of electrochemical properties of NiCo layered double hydroxides via functional pillared effect: An insight into dual charge storage mechanisms. Electrochim. Acta.

[38] Xiong H., Liu L., Fang L., Wu F., Zhang S., Luo H., Tong C., Hu B., Zhou M. (2021). 3D self-supporting heterostructure NiCo-LDH/ZnO/CC electrode for flexible high-performance supercapacitor. J. Alloys Compd..

[39] Hussain I., Hussain T., Yang S., Chen Y., Zhou J., Ma X., Zhang K. (2021). Integration of CuO nanosheets to Zn-Ni-Co oxide nanowire arrays for energy storage applications. Chem. Eng. J..

[40] Mai L.Q., Minhas-Khan A., Tian X., Hercule K.M., Zhao Y.L., Lin X., Xu X. (2013). Synergistic interaction between redox-active electrolyte and binder-free functionalized carbon for ultrahigh supercapacitor performance. Nat. Commun..

[41] Hazra A., Jan A., Tripathi A., Kundu S., Boppidi P.K.R., Gangopadhyay S. (2020). Optimized Resistive Switching in TiO_2_ Nanotubes by Modulation of Oxygen Vacancy Through Chemical Reduction. IEEE Trans. Electron Devices.

[42] Gakhar T., Hazra A. (2020). Oxygen vacancy modulation of titania nanotubes by cathodic polarization and chemical reduction routes for efficient detection of volatile organic compounds. Nanoscale.

[43] Yan D., Li Y., Huo J., Chen R., Dai L., Wang S. (2017). Defect Chemistry of Nonprecious-Metal Electrocatalysts for Oxygen Reactions. Adv. Mater..

[44] Zhang J.J., Wang H.H., Zhao T.J., Zhang K.X., Wei X., Jiang Z.D., Hirano S.I., Li X.H., Chen J.S. (2017). Oxygen Vacancy Engineering of Co_3_O_4_ Nanocrystals through Coupling with Metal Support for Water Oxidation. ChemSusChem.

[45] Li L., Zhang B., Wang S., Fan F., Chen J., Li Y., Fu Y. (2021). Bimetallic NiCo metal-organic framework-derived hierarchical spinel NiCo_2_O_4_ microflowers for efficient non-enzymatic glucose sensing. Bull. Chem. Soc. Jpn..

[46] Liu L., Fang L., Wu F., Hu J., Zhang S., Luo H., Hu B., Zhou M. (2020). Self-supported core-shell heterostructure MnO_2_/NiCo-LDH composite for flexible high-performance supercapacitor. J. Alloys Compd..

[47] Gao X., Liu X., Wu D., Qian B., Kou Z., Pan Z., Pang Y., Miao L., Wang J. (2019). Significant Role of Al in Ternary Layered Double Hydroxides for Enhancing Electrochemical Performance of Flexible Asymmetric Supercapacitor. Adv. Funct. Mater..

[48] Zhang Y., Du D., Li X., Sun H., Li L., Bai P., Xing W., Xue Q., Yan Z. (2017). Electrostatic Self-Assembly of Sandwich-Like CoAl-LDH/Polypyrrole/Graphene Nanocomposites with Enhanced Capacitive Performance. ACS Appl. Mater. Interfaces.

[49] Li Y., Li Q., Zhao S., Chen C., Zhou J., Tao K., Han L. (2018). Conductive 2D Metal-Organic Frameworks Decorated on Layered Double Hydroxides Nanoflower Surface for High-Performance Supercapacitor. ChemistrySelect.

[50] Bai X., Liu Q., Zhang H., Liu J., Li Z., Jing X., Yuan Y., Liu L., Wang J. (2016). Nickel-Cobalt Layered Double Hydroxide Nanowires on Three Dimensional Graphene Nickel Foam for High Performance Asymmetric Supercapacitors. Electrochim. Acta.

[51] Li J., Wei M., Chu W., Wang N. (2017). High-stable α-phase NiCo double hydroxide microspheres via microwave synthesis for supercapacitor electrode materials. Chem. Eng. J..

[52] Li H., Musharavati F., Zalenezhad E., Chen X., Hui K.N., Hui K.S. (2018). Electrodeposited NiCo layered double hydroxides on titanium carbide as a binder-free electrode for supercapacitors. Electrochim. Acta.

[53] Yang P., Jing C., Liu J.C., Chen K., Zhang Y.X. (2020). Controllable crystal growth of a NiCo-LDH nanostructure anchored onto KCu_7_S_4_ nanowires: Via a facile solvothermal method for supercapacitor application. CrystEngComm.

[54] Guan X., Huang M., Yang L., Wang G., Guan X. (2019). Facial design and synthesis of CoSx/Ni-Co LDH nanocages with rhombic dodecahedral structure for high-performance asymmetric supercapacitors. Chem. Eng. J..

[55] Guan T., Fang L., Liu L., Wu F., Lu Y., Luo H., Hu J., Hu B., Zhou M. (2019). Self-supported ultrathin NiCo-LDH nanosheet array/Ag nanowire binder-free composite electrode for high-performance supercapacitor. J. Alloys Compd..

[56] Qiu H., Sun X., An S., Lan D., Cui J., Zhang Y., He W. (2020). Microwave synthesis of histidine-functionalized graphene quantum dots/Ni-Co LDH with flower ball structure for supercapacitor. J. Colloid Interface Sci..

[57] Liu L., Guan T., Fang L., Wu F., Lu Y., Luo H., Song X., Zhou M., Hu B., Wei D. (2018). Self-supported 3D NiCo-LDH/Gr composite nanosheets array electrode for high-performance supercapacitor. J. Alloys Compd..

[58] Zang Y., Luo H., Zhang H., Xue H. (2021). Polypyrrole nanotube-interconnected NiCo-LDH nanocages derived by ZIF-67 for supercapacitors. ACS Appl. Energy Mater..

