# Supplementary material for: Hydrazine Hydrate Induced Three-Dimensional Interconnected Porous Flower-like 3D-NiCo-SDBS-LDH Microspheres for High-Performance Supercapacitor
Source: Materials (Basel). 2022 Feb 14;15(4):1405. doi: 10.3390/ma15041405 (PMC8875902; doi:10.3390/ma15041405)
Supplement: Supplementary file 1 [file materials-15-01405-s001.zip › materials-1563128-supplementary.pdf]

Supplementary Materials for

Hydrazine Hydrate Induced Three-Dimensional Interconnected Porous Flower-like 3D-Nico-SDBS-LDH Microspheres for High-Performance Supercapacitor

Liping Zhong [1], Zumiao Yan [1], Hai Wang [2] and Linjiang Wang [1,3,*]

1 College of Material Science and Engineering, Guilin University of Technology, Guilin 541004, China; zlp19891230@glut.edu.cn (L.Z.); yzm1592022@163.com (Z.Y.)
2 College of Physics and Technology, Guangxi Normal University, Guilin 541004, China; hbwanghai@gmail.com
3 Collaborative Innovation Center for Exploration of Hidden Nonferrous Metal Deposits and Development of New Materials in Guangxi, Guilin University of Technology, Guilin 541004, China
* Correspondence: wlinjiang@163.com

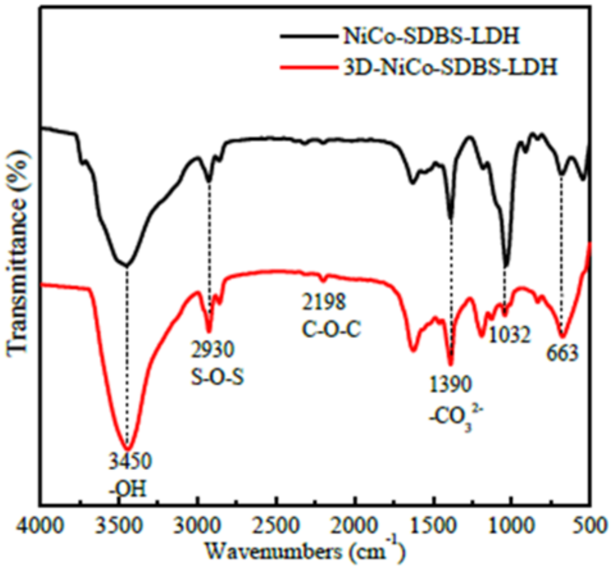

Figure S1. FTIR spectra of NiCo-SDBS-LDH and 3D-NiCo-SDBS-LDH

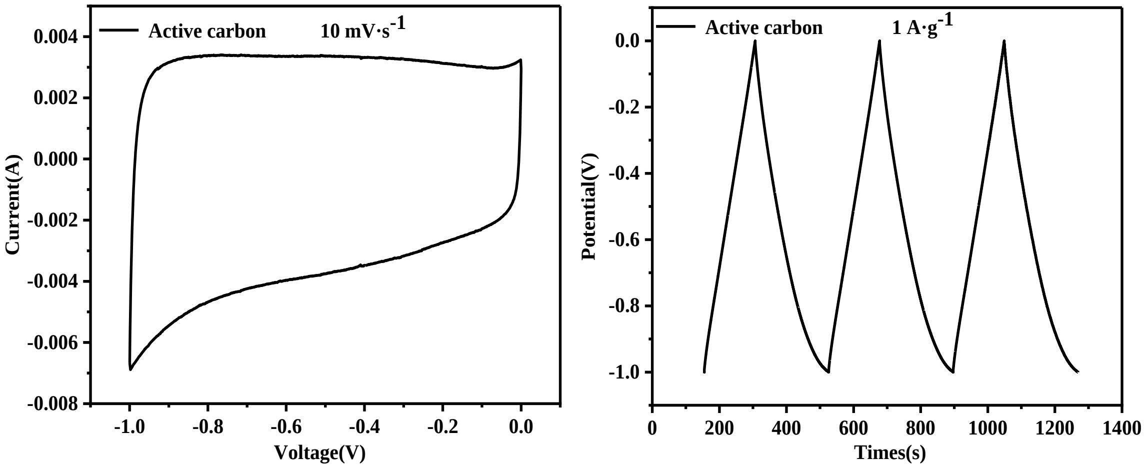

Figure S2. CV curves at 10 mV·s^{-1} and GCD curves at 1A·g^{-1} of activated carbon.
